# How does age affect the care dependency risk one year after stroke? A study based on claims data from a German health insurance fund

**DOI:** 10.1186/s12877-015-0130-0

**Published:** 2015-10-23

**Authors:** Susanne Schnitzer, Olaf von dem Knesebeck, Martin Kohler, Dirk Peschke, Adelheid Kuhlmey, Liane Schenk

**Affiliations:** Department of Medical Sociology and Rehabilitation Science, Charité–Universitätmedizin Berlin, Luisenstr. 57, D-10117 Berlin, Germany; Department of Medical Sociology, University Medical Center Hamburg-Eppendorf, Martinistr. 52, D-20246 Hamburg, Germany; Central Research Institute of Ambulatory Health Care in Germany, Herbert-Lewin-Platz 3, D-10623 Berlin, Germany; Department of Structural Advancement and Quality Management in Health Care, Technische Universität Berlin, Steinplatz 2, D-10623 Berlin, Germany

**Keywords:** Age, Stroke, Post-stroke care dependency, Geriatric multimorbidity

## Abstract

**Background:**

The objective of this study is to investigate the effect of age on care dependency risk 1 year after stroke. Two research questions are addressed: (1) How strong is the association between age and care dependency risk 1 year after stroke and (2) can this association be explained by burden of disease?

**Methods:**

The study is based on claims data from a German statutory health insurance fund. The study population was drawn from all continuously insured members with principal diagnoses of ischaemic stroke, hemorrhagic stroke, or transient ischaemic attack in 2007 who survived for 1 year after stroke and who were not dependent on care before their first stroke (*n* = 2864). Data were collected over a 1-year period. People are considered to be dependent on care if they, due to a physical, mental or psychological illness or disability, require substantial assistance in carrying out activities of daily living for a period of at least 6 months. Burden of disease was assessed by stroke subtype, history of stroke, comorbidities as well as geriatric multimorbidity. Regression models were used for data analysis.

**Results:**

21.6 % of patients became care dependent during the observation period. Post-stroke care dependency risk was significantly associated with age. Relative to the reference group (0–65 years), the odds ratio of care dependency was 11.30 (95 % CI: 7.82–16.34) in patients aged 86+ years and 5.10 (95 % CI: 3.88–6.71) in patients aged 76–85 years. These associations were not explained by burden of disease. On the contrary, age effects became stronger when burden of disease was included in the regression model (by between 1.1 and 28 %).

**Conclusions:**

Our results show that age has an effect on care dependency risk that cannot be explained by burden of disease. Thus, there must be other underlying age-dependent factors that account for the remaining age effects (e.g., social conditions). Further studies are needed to explore the causes of the strong age effects observed.

## Background

As the number of older people in western populations rises, stroke prevalence is expected to increase [1]. In both men and women, stroke rates increase exponentially with age [2]. Stroke is a main cause of disability and care dependency in adults [3, 4], and age is also known to play an important role in post-stroke outcomes. Glader et al. analysed data from 19,547 patients included in the Swedish National Quality Register for Stroke Care [5]. Their results show that, among patients who lived at home before their stroke, age was a strong predictor for living in institutional care 3 months after stroke—with and without control for other variables. In a study of patients recovering from stroke in a long-term rehabilitation hospital, Koyama et al. [6] found that older age increased the odds of discharge to a nursing home rather than directly to the patient’s home. Their correlation analyses further revealed that older age was associated with female sex, ischaemic stroke, lower scores on the Functional Independence Measure (FIM-m), smaller household size, and a higher number of sons/daughters.

As pointed out by Hankey et al. [7], although advancing age is known to be strongly associated with increasing levels of disability and an increasing number of comorbidities, few studies have examined the independent effect of age on post-stroke outcomes. Irrespective of stroke, the burden of comorbidity is known to be substantial in older adults. In Germany, it is assumed that one in three adults aged over 70 years has five moderately severe conditions and that almost one in four is in treatment for five conditions concurrently. In the German Ageing Survey 2002, 24 % of respondents aged 70+ years reported that they suffered from five or more conditions, whereas only 7 % did not report any conditions at all [8]. Stroke survivors are often affected by stroke-related conditions such as aphasia and hemiparesis. Thus, it could be assumed that older stroke patients are at a higher risk of becoming care dependent because they have more comorbidities.

Consequently, in our study we addressed two research questions: (1) How strong is the association between age and care dependency risk 1 year after stroke? (2) Can this association be (partly) explained by burden of disease?

## Methods

### Study design and sample

The study is based on claims data, which collates information on inpatient hospital treatment and outpatient care in compliance with its statutory mandate. These data were complemented by information on members’ age, gender, and date of death. The study population was drawn from a sample comprising all continuously insured members of the Deutsche BKK who received acute inpatient care for cerebrovascular disease in 2007 (ICD-10-GM: I60–I69 and G45) (*N* = 5599). For the following analyses, the sample was restricted to insurants with a principal diagnosis of ischaemic stroke (ICD-10-GM: 163), hemorrhagic stroke (ICD-10-GM: I60, I61) or transient ischaemic attack (TIA) (ICD-10-GM: G45) (*n* = 4464). All data were pseudonymized. Ethical approval was not required in view of the nonexperimental design of the study. The observation period covered the first year after admittance to acute inpatient care. The data collection period ended on December 31, 2008. Patients who died during the observation period and patients who were already dependent on care before their first stroke were excluded. This reduced the dataset to 2864 stroke survivors. The data used were provided by the German company health insurance funds (Deutsche BKK), who granted the author permission to use it.

### Measures

We defined patients as dependent on care if they were eligible for long-term care benefits in accordance with the German Social Code at any time during the period of observation and had thus been assessed by qualified personnel according to a standardized procedure specified by law. The decisive factor here is the time frame over which assistance is needed, irrespective of where the recipient lives (at home or in a nursing home). People are considered to be dependent on care if they, due to a physical, mental or psychological illness or disability, require substantial assistance in carrying out activities of daily living for a period of at least 6 months. The level of care benefits provided depends on the degree of impairment [9]. Data were available on care status at the beginning of the period of observation as well as on the last change in care status (and the date thereof). The data were processed to allow us to distinguish between patients who required long-term care before their stroke from those who did not.

To ascertain the care dependency risk, we created a dichotomous variable (0: no care, 1: care) distinguishing patients who were not dependent on care at any time from those who became dependent on care over the period of observation (*n* = 617). We assessed burden of disease by reference to the following data: stroke subtype (ischaemic, hemorrhagic, TIA); history of stroke in the previous year; comorbidities (aphasia,hemiparesis, dysphagia, diabetes, chronic kidney insufficiency or heart failure) and geriatric multimorbidity. The methods for compiling the list of symptom complexes characterizing geriatric patients and the corresponding ICD-10 codes have been described in detail elsewhere [10, 11]. In short, geriatric multimorbidity was classified according to the diagnoses known at the time of hospital discharge, based on the catalogue of the German Society of Geriatrics (DGG) – Diagnosis Related Groups (DRG). Geriatric multimorbidity covers the following symptom complexes: frailty, urinary incontinence, immobility, cognitive impairment, falls risk, pressure ulcer, malnutrition, disorders of fluid and electrolyte balance, depression, pain, neuropathies, severe visual disturbances and hearing loss, medication problems, high risk of complications and delayed convalescence. Appendix shows the geriatric symptom complexes according to their ICD-10 codes and the frequency of the different complexes in our study population. The number of these diagnoses was ascertained for each insured person, and a geriatric multimorbidity indicator of 0, 1, 2, 3, or 4+ morbidities was assigned accordingly. Consistent with the DGG–DRG catalogue, we defined geriatric multimorbidity as the presence of two or more of these geriatric diagnoses in the same individual. We use the term comorbidities to subsume diagnoses resulting from the stroke event (i.e., aphasia, dysphagia and hemiparesis) and diagnoses which are associated with a stroke event (i.e., diabetes, chronic kidney insufficiency or heart failure). These definitions are in line with the widely used terms “comorbidity” and “multimorbidity”. Comorbidity refers to a combination of additional diseases beyond an index disorder—here, the focus of interest is on an index condition and the possible effects of other disorders on its prognosis. In contrast, multimorbidity is defined as any co-occurrence of diseases in the same person—the focus of interest here is on individuals who suffer from multiple diseases rather than on a given index condition [12].

### Statistical analyses

Chi-square test or Mann-Whitney U test used to test group differences presented in Table 1. Binary logistic regression models were used to explore the association between care dependency risk and age and to ascertain the interdependence between care dependency, age and burden of disease. The stepwise forward procedure was chosen to detect unadjusted and adjusted age effects and to determine associations between the explanatory factors. We considered five models. In a first step, we analysed the univariate association between care dependency risk and age (Model 1); in a second step, we included gender in the model to explore gender-specific differences in age effects (Model 2). In a third step, burden of disease was entered in four further models: Model 3 incorporated history of stroke in the previous year, Model 4 included stroke subtype, Model 5 integrated comorbidities (see above), and geriatric multimorbidity was entered in Model 6. In Models 2, 3, 4 and 6, the variables were included using the standard procedure (adjusted for all included variables). In Model 5, we used the forward Wald procedure to guide model entry and enter only those comorbidities that showed significant effects. In a fourth and final step, we included interaction terms between age and stroke subtype, and between age and gender, as conflicting trends were suspected here (e.g., high care dependency risk for elderly women/young men). As these interactions were not significant, we did not include them in the final model (Model 6). The degree to which the association between care dependency risk and age was explained by the other factors outlined above was assessed by the percentage change in the odds ratios (OR) when those other factors were introduced in the Models 2 to 6. The results are shown in Table 2 as OR with 95 % confidence intervals. All statistical analyses were performed using SPSS 19.

**Table 1:** Burden of disease and gender based on age groups

	Total	0–65 years	66–75 years	76–85 years	86+ years	*p*
	2864 (*n*)	850 (*n*)	938 (*n*)	884 (*n*)	192 (*n*)	
Gender						<0.001
Men	45.2	57.8	48.4	33.4	28.6	
Women	54.8	42.2	51.6	66.6	71.4	
Stroke subtype						<0.001
TIA	32.8	33.8	33.2	30.9	35.9	
Ischaemic	58.9	53.9	59.6	63.2	58.3	
Hemor.	8.2	12.4	7.2	5.9	5.7	
Stroke previous year						
Yes	6.3	4.7	6.5	7.4	7.8	0.11
Geriatric Multimorbidity						<0.001
0 and 1	75.7	83.5	79.6	66.3	65.6	
2	15.5	11.3	14.1	20.2	18.8	
3	6.0	3.5	4.6	8.7	11.5	
4+	2.8	1.6	1.7	4.8	4.2	
Diagnoses						
Aphasia	31.8	24.5	29.9	39.1	39.6	<0.001
Dysphagia	7.0	5.6	5.8	9.5	7.8	<0.005
Hemiparesis	35.8	31.9	36.6	38.8	34.9	<0.05
Diabetes	23.6	16.1	27.1	27.1	23.4	<0.001
Chronic kidney insufficiency	6.0	2.1	5.5	8.8	13.0	<0.001
Heart failure	6.8	2.8	5.0	11.4	12.0	<0.001
Care						
Yes–in observation period	21.6	8.9	15.6	33.4	52.6	<0.001

All figures in percent unless otherwise indicated (*n*)

**Table 2:** Association between age and care dependency at one year after stroke: results of multivariate prediction models, stepwise adjusted for burden of disease (*N* = 2864)

	Model 1	Model 2		Model 3		Model 4		Model 5		Model 6	
Nagelkerke R Square	14.0 %	14.1 %		20.6 %		31.3 %		34.5 %			
	OR^a^ (CI)	OR^a^ (CI)	Change^b^	OR^a^ (CI)	Change^b^	OR^a^ (CI)	Change^b^	OR^a^ (CI)	Change^b^		Change^b^
Age											
0–65 years	1	1		1		1		1		1	
66–75 years	1.88 (1.40–2.52)	1.85 (1.38–2.49)	−1.6 %	1.82 (1.36–2.45)	−3.2 %	1.96 (1.45–2.65)	+4.3 %	1.92 (1.38–2.65)	+2.1 %	1.90 (1.36–2.65)	+1.1 %
76–85 years	5.10 (3.88–6.71)	4.94 (3.74–6.52)	−3.1 %	4.84 (3.67–6.40)	−5.1 %	5.38 (4.03–7.18)	+5.5 %	5.28 (3.86–7.21)	+3.5 %	4.77 (3.47–6.57)	−6.5 %
86 + years	11.30 (7.82–16.34)	10.88 (7.50–15.78)	−3.7 %	10.70 (7.37–15.55)	−5.3 %	13.17 (8.92–19.45)	+16.5 %	14.46 (9.58–21.81)	+28 %	13.30 (8.74–20.24)	+17.7 %
Gender											
Women		1.15 (0.94–1.40)		1.18 (0.97–1.43)	+2.6 %	1.24 (1.01–1.52)	+7.8 %	1.24 (1.00–1.53)	+7.8 %	1.14 (0.92–1.43)	−0.87 %
Stroke previous year											
Yes		–		2.05 (1.46–2.87)		2.18 (1.54–3.09)	+0.1 %	1.95 (1.35–2.83)	−4.8 %	1.75 (1.19–2.55)	−14.6 %
Stroke subtype											
TIA		–				1		1		1	
Ischaemic		–				2.8 (2.20–3.57)		1.59 (1.21–2.07)	−43.2 %	1.48 (1.13–1.95)	−47.1 %
Hemor.		–				6.01 (4.16–8.67)		3.39 (2.28–5.03)	−43.6 %	2.92 (1.95–4.37)	−51.4 %
Diagnoses											
Aphasia		–				–		1.39 (1.12–1.74)		1.41 (1.13–1.77)	+1.4 %
Dysphagia		–				–		6.83 (4.70–9.90)		5.34 (3.63–7.86)	−21.8 %
Hemiparesis		–				–		1.82 (1.44–2.28)		1.85 (1.46–2.33)	+1.65 %
Diabetes		–				–		1.50 (1.19–1.88)		1.43 (1.13–1.80)	−4.7 %
Geriatric multi-morbidity											
0 and 1		–				–		–	–	1	
2		–				–		–	–	2.35 (1.81–3.04)	
3		–				–		–	–	3.08 (2.13–4.47)	
4+		–				–		–	–	3.53 (2.04–6.10)	

^a^ *OR* Odds Ratio, *CI* Confidence Interval

^b^Percentage change in coefficient; first included predictors (reference model) compared separately at a time with the other models

## Results

### Characteristics of the study sample

Most of the stroke patients in our sample fell into the 66–85 age group (Table 1). Overall, women outnumbered men (54.8 versus 45.2 %), with the exception of the youngest age group (0–65 years), in which more men (57.8 %) than women (42.2 %) had received acute inpatient care for stroke. Notably, there were more haemorrhage patients in this youngest age group (12.4 %) than in the other age groups (5.7–7.2 %; most fatalities occurred in the older age groups). 24.3 % of patients were classified as affected by geriatric multimorbidity (i.e., had two or more associated conditions). Around one-third had secondary diagnoses of hemiparesis (35.8 %) or aphasia (31.8 %). 21.6 % became care dependent during the observation period (*n* = 618).

### Associations between care dependency risk and age

Age was significantly associated with care dependency risk in all models (Table 2, Models 1–6). Relative to the reference group (0–65 years), the odds ratio of care dependency was 11.30 (95 % CI: 7.82–16.34) in patients aged 86+ years and 5.10 (95 % CI: 3.88–6.71) in patients aged 76–85 years (Model 1). When gender was controlled for (Model 2), there was a reduction of between 1.6 and 3.7 % in the odds ratio for age, but gender itself had no significant effect on care dependency risk. When history of stroke in the previous year was included (Model 3), the odds ratio for age decreased by 3.2 to 5.3 %, but with the inclusion of stroke subtype (Model 4) there was an increase in the odds ratio for age (4.3–16.5 %). The same held in Models 5 and 6, when comorbidities were introduced: the odds ratios for age increased by 1.1 to 28 %. By contrast, the odds ratios for stroke subtype and prior stroke event decreased by 4.8 to 51.4 % when comorbidities were included (Models 5–6). Besides age, the final model revealed a particularly strong association of care dependency risk with dysphagia (OR: 5.34; 95 % CI: 3.63–7.86) and geriatric multimorbidity. Relative to the reference group, patients with four or more geriatric morbidities were 3.5 times more likely to be dependent on care 1 year after stroke (OR: 3.53; 95 % CI: 2.04–6.10).

## Discussion

This study analysed associations between age and care dependency risk after stroke. Together with findings from other studies, our results indicate increasing care dependency rates with older age [4, 6, 7, 9]. Relative to the reference group (0–65 years), patients aged 86+ years were 11.30 times more likely (95 % CI: 7.82–16.34) to be care dependent 1 year after stroke, and patients aged 76–85 years were 5.10 times more likely (95 % CI: 3.88–6.71). Even after the stepwise inclusion of gender, stroke subtype, history of stroke in the previous year and comorbidities, these age effects remained largely stable. In other words, the care dependency risk increases with age, irrespective of the presence of all other variables. Only the inclusion of history of stroke in the previous year and gender minimally reduced the effect of age. Age-adjusted gender was not associated with care dependency risk after stroke. Several studies investigating gender-specific differences in stroke care have attributed these differences to female patients’ more advanced age [4, 13]. Other age-adjusted studies have also found that gender is not significantly associated with further disease progression after stroke [14, 15].

Furthermore, we examined whether age effects could be explained by burden of disease. It is well known that older people typically have several chronic conditions concurrently [7, 8] and that the prevalence of multimorbidity increases substantially with age [16]. Multimorbidity affects more than half the elderly population, with increasing prevalence in very old persons [12]. At the same time, consistent with our results, studies suggest an association between post-stroke care dependency and burden of disease. A large European study of hospital admissions for acute stroke found that swallowing problems and urinary incontinence were significantly related to disability and handicap 3 months after stroke [17]. Other studies have found stroke subtype, prior stroke event or pre-stroke disability to have a significant effect on care dependency risk [4, 6, 7, 18]. Against this background, we hypothesised that the association between age and care dependency risk the first year after stroke would be mediated by burden of disease. However, the results of our stepwise regression analyses revealed that only with the inclusion of prior stroke events the odds ratios of age were reduced minimally. Apart from this result, age associations were not reduced after adjustment for burden of disease. On the contrary, inclusion of both comorbidities and geriatric mulitmorbidity increased the effect of age on care dependency risk (by between 1.1 and 28 %).

This result raises a question of which other factors may account for the remaining age effects. It seems probable that social factors such as living in a partnership or alone, social contacts or social support may play a role. As several studies have reported associations between social factors and care dependency risk and between age and social factors, it seems likely that social factors mediate the association between care dependency risk and age. Appelros et al. [18] examined the association between living setting and need for assistance with activities of daily living before and 1 year after a first-ever stroke. Living alone at baseline increased the odds of living in a service flat or nursing home 1 year after stroke 2.7-fold (95 % CI:1.4–5.1). The authors concluded that this finding to some extent could be attributed to the fact that there are more single women than single men: Spouses take great responsibility for helping their partners after a stroke, and males receive more personal help from their female spouses than vice versa. By the same token, Koyama et al. [6] found that patients without a spouse at home who lived in smaller households were more likely to be discharged to a nursing home after a stroke. Furthermore, as mentioned above, they found that age was negatively correlated with the number of household members [6]. To sum up, social conditions such as living together with a spouse are protective factors regarding (post-stroke) care dependency risk. As older age leads to a loss of social contacts and partnerships, it seems likely that the association of age with care dependency after stroke found in our study may be partly explained by such social factors. Unfortunately, we were not able to investigate the effects of social contacts and partnerships on care dependency risk after stroke, as the health insurance data did not include this information for data protection reasons.

Other possible explanations for the strong age effects revealed in our study are neurochemical and physiological changes in older age. Although it is well known that vulnerability for negative outcomes increases with age due to a decreasing reserve of the physiological systems, little is known about the underlying age-dependent mechanisms. Findings from a recent study indicate that hormones modulate the age-dependent differential stroke outcomes [19]. Further studies are needed to investigate whether such neurochemical effects associated with age also increase the risk of becoming care dependent (after stroke).

Our results also show that geriatric multimorbidity has a significant effect on post-stroke care dependency risk. This finding has clear practical implications, because there is potential to influence the progression and extent of comorbidities. Internationally, treatment in a specialized interdisciplinary stroke unit, followed by inpatient rehabilitation, is the gold standard [20]. Outpatient services are also essential in safeguarding continuity of care and mitigating the long-term effects of stroke. However, in Europe, including Germany, outpatient rehabilitation is underutilized [20]. The provision of in- and outpatient rehabilitation services should therefore be increased to reduce stroke-associated comorbidities and thus decrease the post-stroke care dependency risk. Further studies are needed to explore the pattern and characteristics of geriatric morbidities in more detail.

The limitations of our study include the lack of data on social factors in the data set used. Moreover, it was not possible to differentiate between comorbidities that were already present before the stroke and those that were caused by the stroke. Furthermore, it is questionable whether the list of symptom complexes characterizing geriatric multimorbidity covers all aspects of disease burden; we cannot rule out the possibility that the number of diagnoses was underestimated. Divergences from the findings of other studies may also be attributable to differences in methods of data assessment: the diagnoses were ascertained from claims data and not made by specially trained personnel in the context of a primary data assessment. However, the approach to assess geriatric multimorbidity applied by the German Geriatric Association has proved to be generally practicable and successful [21]. Finally, it remains unclear to what extent the present data can be considered representative of the general German population. The Deutsche BKK is a German company health insurance fund, with around 1.1 million members [22]. Previous analyses of the Deutsche BKK data have shown nearly the same distribution of the stroke subtypes as reported in other sources (e.g., regional stroke registers and, in particular, national DRG data). Regarding sex distribution, however, women in the age groups older than 60 years have been found to be overrepresented [23]. The company health insurance funds have been found to provide among the best representations of the German population with respect to socio-demographic indicators such as age distribution orEast/West ratio [24].

We used the standard definition of care dependency applied in Germany, i.e., the patient was receiving long-term benefits from their care insurer. Although this definition is not in line with major international operational definitions of care dependency, the prevalence identified in our study (17.5 %) is similar to other studies. In international studies, the onset of care dependency is defined in terms of the degree of physical impairment (modified Rankin Scale), reduced independence (Barthel Index) or place of residence (at home, at home with carers’ support, nursing home). The available data are mainly drawn from retrospective surveys. The findings of these studies indicate that 15–19 % of previously independent persons are so impaired by stroke that they are reliant on others’ help for daily living (long-term care) [18, 13, 25].

The strengths of our study include the fact that claims data are not subject to non-response. This is a major advantage when investigating populations with a high proportion of people with limited communication abilities. Use of claims data also excludes memory errors, which is particularly important in studies with very old respondents. Moreover, the study design excludes the possibility of institution-related selection bias and drop-out at follow-up. As such, the data examined are guaranteed to be complete [26].

## Conclusions

To our best knowledge, this is the first study to analyse factors that may account for the strong association between (post-stroke) care dependency and age. Our results show that age has an independent effect on care dependency risk that is not explained by burden of disease. Further research is needed to determine the causes of these strong age effects.

## Appendix

### Frequency of symptom complexes characterizing geriatric patients and their corresponding ICD-10 codes used in this study [11]

**Table:**

Symptom complexes characterizing geriatric patients	ICD-10 codes	*n* (%)
Immobility	M96.8, M62.3, M62.5	10 (0.3)
Falls risk and dizziness	R26, R29.81, R42, H81, H82	473 (16.5)
Cognitive impairment	G30, F00, F01, F02-G20, F04, F05, F06.7, F07.0-F07.2, F.07.8	168 (5.9)
Incontinence	R32, N39.3, N39.4, R15	417 (14.6)
Pressure ulcer	L89, L97, 183.0, 183.2, L98.4	51 (1.8)
Malnutrition	R64, E41, E43, E44	4 (0.1)
Disorders of fluid and electrolyte balance	E86, E87, R60	285 (10.0)
Depression and anxiety	F32, F33, F30, F31, F40, F41	141 (4.9)
Pain	R52, R51, N23, R10, M54, K08.88, F62.80, H57.1, M79.6, M25.5, R07.0-R07.4, N64.4, H92.0, F45.4, M75.8, K14.6	20 (0.7)
Neuropathies	R20, G50-G59, G60-G64	350 (12.2)
Frailty	R54	20 (0.7)
Severe visual disturbances and hearing loss	H53, H54, H52.4, H25, H28, H90, H91	205 (7.2)
Medication problems	Y57.9, X49.9	12 (0.4)
High risk of complications	Z98, Z48, Z43, T79-T89, Z99.2, I48	561 (19.6)
Delayed convalescence	Z54	1 (0.03)

## Abbreviations

BKK: Betriebskrankenkasse [company health insurance fund]
ICD: International Statistical Classification of Diseases and Related Health Problems
DGG: Deutsche Gesellschaft für Geriatrie [German Society of Geriatrics]
DRG: Diagnosis Related Groups
TIA: Transient ischaemic attack
OR: Odds ratios
CI: Confidence interval
OP: Observation period
